# Drought diminishes ecosystem service supply and exacerbates trade-offs in the Yangtze River Economic Belt

**DOI:** 10.1016/j.isci.2025.113604

**Published:** 2025-09-19

**Authors:** Liujie He, Shuyang Wu, Zeyang Xie, Han Liang, Zhijian Wu, Deli Xiao, Jinqi Zhu, Bofu Zheng, Wei Wan

**Affiliations:** 1 School of Resources & Environment, Nanchang University, Nanchang 330031, China; 2 Engineering Research Center of Watershed Carbon Neutralization, Key Laboratory of Poyang Lake Environment and Resources Utilization, Ministry of Education, Jiangxi Institute of Ecological Civilization, School of Resources and Environment, Nanchang University, Nanchang 330031, China; 3 School of Infrastructure Engineering, Nanchang University, Nanchang 330031, China

**Keywords:** Environmental science, Environmental analysis, Environmental resource

## Abstract

Climate change has intensified drought frequency globally, driving the need to assess its impacts on ecosystem services (ESs) for sustainability planning. This study investigated meteorological drought thresholds and ES interactions in China’s Yangtze River Economic Belt. Results demonstrated that extreme droughts reduced ES provisioning capacity, with significant trade-offs between water retention and food supply (*R* = −0.25, *p* < 0.001), and between soil conservation and food supply (*R* = −0.32, *p* < 0.001). Water retention and soil conservation were more susceptible to drought, whereas carbon sequestration was less affected. Constraint line analysis revealed that ES thresholds predominantly occurred under non-drought conditions. Mechanistic analyses found that under high drought loadings, climatic factors exerted a significant negative impact on ESs. This study provides a scientific basis for regional drought adaptation strategies and the coordinated management of ESs, offering references for optimizing land use patterns and enhancing ecosystem sustainability under climate change.

## Highlights

• Constraint thresholds between SPEI and ESs were identified
• Forest WR and SC were more vulnerable to meteorological drought
• Trade-offs between WR and FS, SC, and FS were strongest in extreme drought areas
• Revealed the coupling effects between drought and other drivers

## Graphical abstract

**Figure:**
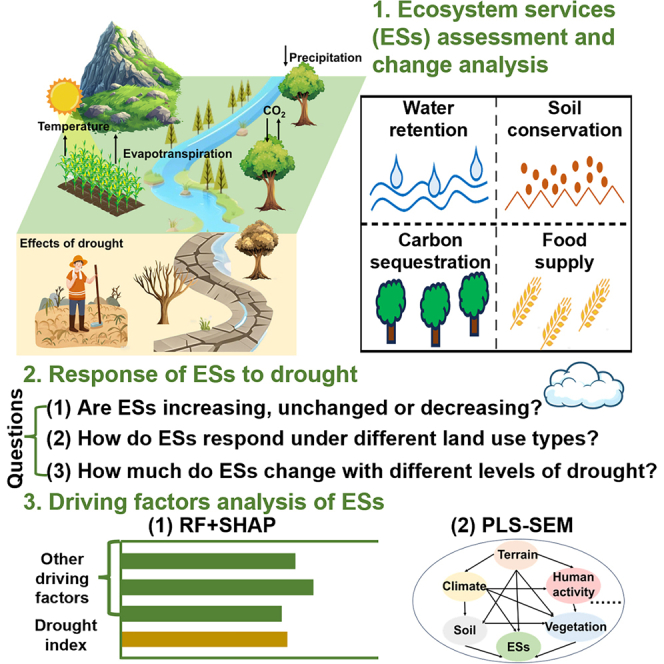

## Introduction

The intensification of global warming has markedly accelerated terrestrial water cycle processes,^1^ with the global land area affected by prolonged droughts expanding at 49,279 ± 14,771 km^2^ annually.^2^ Under persistent future warming scenarios, droughts are projected to become more frequent, prolonged, and severe,^3^ posing substantial threats to ecosystems and human societies.^4^ Over the past five decades, droughts have caused approximately 650,000 deaths and $124 billion in global economic losses.^5^ Drought refers to water resource scarcity caused by imbalances in hydrological budgets or supply-demand dynamics,^6^ primarily categorized into meteorological drought, hydrological drought, agricultural drought, and socioeconomic drought.^7^ In terrestrial ecosystems, drought primarily alters ecohydrological processes within the soil-plant-atmosphere continuum, thereby influencing material cycles and energy flows.^8^ Nevertheless, ecosystems possess inherent resistance capacities to maintain stability under moderate disturbances, including resilience to drought impacts.^9^ Consequently, understanding ecosystem responses and adaptations to climate change has emerged as a critical scientific issue in related disciplines.

Ecosystem services (ESs) refer to the diverse benefits humans obtain from ecosystems, encompassing ecological characteristics, functions, and processes that directly or indirectly enhance human well-being,^10^ which are vital for human survival and development.^11^ Studies demonstrate that drought negatively impacts ESs,^12^ leading to biodiversity loss, reduced carbon storage, and diminished water conservation capacity.^13^^,^^14^ Morán-Ordóñez et al.^15^ projected that future droughts might decrease forest provisioning and regulating services by approximately 4.43% and 8.6%, respectively. However, other studies indicate that particular ecosystems can self-recover, maintain, or even moderately enhance ES supply under mild drought stress.^16^ For instance, North American grasslands showed no significant decline in aboveground productivity with prolonged drought duration,^17^ potentially due to the vegetation regulating water potential and photosynthetic rate through stomatal behavior.^18^ Moreover, the functional diversity of the plant community can resist the negative effects of drought stress on ESs.^19^^,^^20^ Furthermore, drought effects on ESs exhibit complexity and spatial heterogeneity, closely associated with ecosystem type, species diversity, and adaptive capacity. Therefore, elucidating the impacts of drought on ESs and identifying their thresholds forms the foundation for ensuring sustainable ES supply.

Precipitation, temperature, elevation, land use types, and urbanization are considered key drivers that directly or indirectly affect ESs.^21^^,^^22^^,^^23^^,^^24^ As essential climatic manifestations, temperature and precipitation regulate ecosystem composition, structure, and distribution. Precipitation deficits combined with abnormally high temperatures may trigger meteorological drought events, thereby threatening ecosystem stability.^25^^,^^26^ Significant variations in meteorological drought responses exist across land use types.^27^ Research indicates that forest ecosystems in the monsoon-affected temperate and subtropical zones of China exhibit lower drought thresholds, attributed to their enhanced capacity for extracting deep soil water and retaining moisture within root zones, effectively mitigating the adverse impacts of drought.^28^ Under current climate change scenarios, the impacts of increasingly frequent drought events on ESs cannot be overlooked.^29^ Multiple ecosystem structural and functional attributes exhibit abrupt shifts along drought gradients.^30^ However, most current studies focus on the effects of single driving factors on ESs and fail to reveal the coupled effects of drought with other multiple stressors.

The Yangtze River Economic Belt (YREB) serves as both a pivotal engine driving China’s high-quality economic development and a demonstration zone for green development to practice ecological civilization. Under climate change impacts, the YREB has experienced recurrent drought events, most notably during the summer of 2022, when the region endured its most severe extreme drought since 1961,^31^ which caused substantial ecosystem degradation and direct economic losses amounting to 51.28 billion yuan. Sun et al.^32^ project persistent intensification of drought event frequency and magnitude across the Yangtze River Basin under future climate scenarios. Consequently, quantifying drought impacts on YREB’s ESs and elucidating underlying mechanisms are critical for safeguarding a stable ES supply. This study aims to: (1) clarify the spatiotemporal patterns of typical ESs and meteorological drought in the YREB since 2000; (2) decipher gradient responses of ESs to drought across land use types and identify critical thresholds; and (3) systematically assess the contribution of drought to changes in ESs among the drivers and their coupling mechanism. Our results are intended to provide scientific references for regional ecosystem sustainability and the formulation of climate change strategies.

## Results

### Spatiotemporal distribution of ecosystem services and meteorological drought

**Figure 1:**
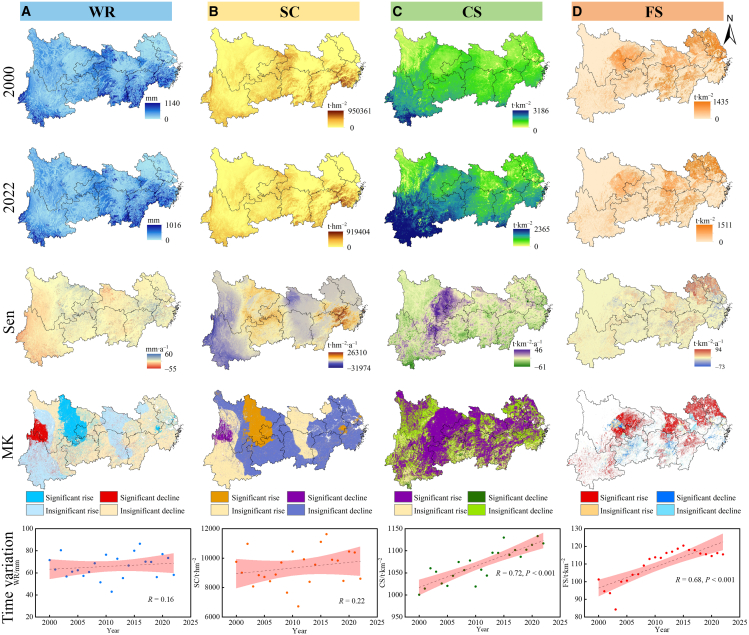
Spatiotemporal dynamics of ESs (A), (B), (C), and (D) show the spatial distribution and spatiotemporal trends of water retention (WR), soil conservation (SC), carbon sequestration (CS), and food supply (FS), respectively, from 2000 to 2022. The shaded areas represent the 95% confidence intervals. MK, Mann-Kendall test; Sen, Theil-Sen median slope estimator. The same below.

The spatial distribution of ESs in the YREB, exhibiting heterogeneous characteristics, is presented in Figure 1. Water retention (WR) and soil conservation (SC) demonstrated similar spatial variation patterns, with their high-value areas predominantly distributed in the mountainous and hilly regions—particularly in southern Yunnan, western Hubei and Hunan, and the hilly areas of Jiangxi and Zhejiang. Carbon sequestration (CS) high-value areas were concentrated in the mountainous regions of Yunnan and Guizhou, whereas food supply (FS) high-value areas were predominantly located in the Sichuan Basin and Middle-Lower Yangtze Plain. CS and FS showed highly significant increasing trends (*p* < 0.001). From 2000 to 2022, CS increased from 1000.48 to 1116.65 t km^−2^, whereas FS increased from 101.23 to 115.45 t km^−2^. Additionally, Figure 1 shows areas with significant decreasing trends in WR and SC at the junction of northern Yunnan Province and western Sichuan Province (*p* < 0.05). We identified areas with significantly increasing trends in CS in eastern Sichuan Province and western Guizhou Province (*p* < 0.05). Areas with significant increasing trends in FS were primarily concentrated in the major grain-producing regions of the YREB (*p* < 0.05). By calculating the average Sen’s slope value for each province (Figure S1), we found that from 2000 to 2022, WR and SC decreased in Yunnan Province, with changing trends of −0.56 mm·a^−1^ and −77.94 t hm^−2^·a^−1^, respectively. In contrast, WR and SC in Zhejiang Province showed relatively large increasing trends, with changing trends of 1.09 mm·a^−1^ and 294.3 t hm^−2^·a^−1^, respectively. CS increased across most areas spatially. FS showed prominent decreasing trends in regions such as Shanghai Municipality and Zhejiang Province, with decreases of −2.55 and −2.15 t km^−2^·a^−1^, respectively.

**Figure 2:**
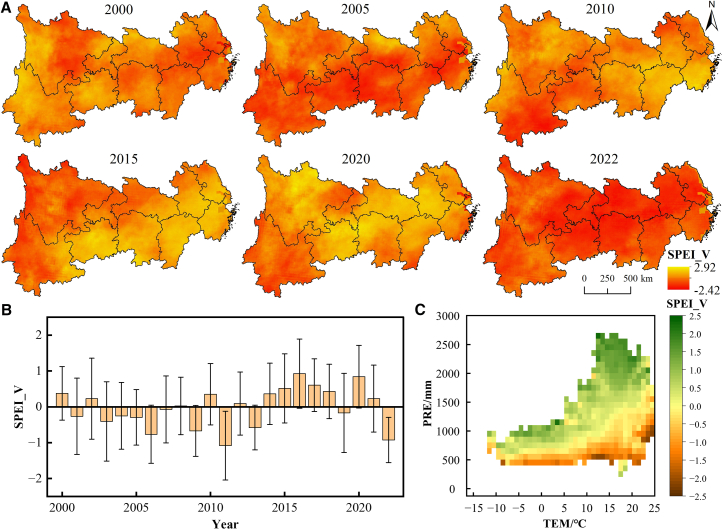
Spatiotemporal characteristics of the SPEI (A) Spatial patterns of SPEI. (B) Temporal evolution of SPEI. Data are represented as mean ± standard deviation (SD). (C) Mean SPEI values across temperature and precipitation ranges. SPEI, standardized precipitation evapotranspiration index; SPEI_V, SPEI value; a smaller SPEI_V indicates a higher severity of drought. The same below.

Figure 2 shows the spatiotemporal variations of the standardized precipitation evapotranspiration index (SPEI) in the YREB, with most regions experiencing their severe meteorological drought in 2022 (Figure 2A). Between 2000 and 2022, the annual average SPEI value remained above −2.5 (Figure 2B), but it dropped notably from 0.35 in 2010 to −1.08 in 2011 and from 0.23 in 2021 to −0.93 in 2022. Statistical analysis identified higher drought probability when annual mean temperatures ranged between 23°C and 25°C or annual precipitation fell within 400–600 mm (Figure 2C).

### Response of ecosystem services to drought under different land use types

**Figure 3:**
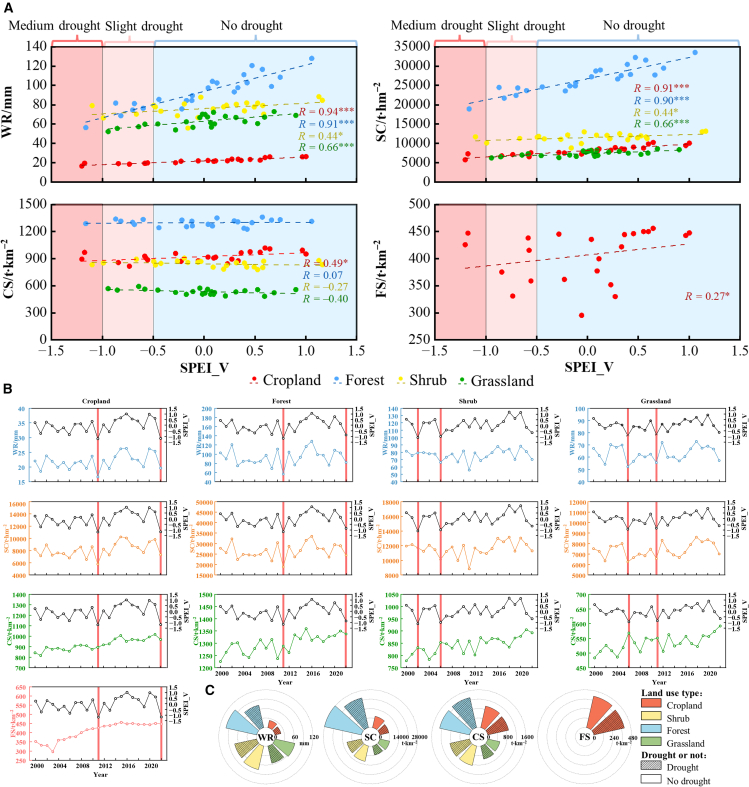
Effects of drought on ESs under different land use types (A) Linear relationship between ESs and SPEI. (B) Temporal change characteristics of ESs and SPEI, where the red vertical line indicates the year of severe drought. (C) Comparative analysis of ES means under drought and non-drought conditions across land use types. ∗ represents a significant level at *p* < 0.05; ∗∗ represents a highly significant level at *p* < 0.01; ∗∗∗ represents an immensely significant level at *p* < 0.001. The same below.

Forests demonstrated stronger capacities in providing multiple regulating services across different land use types (Figure 3). Under drought conditions, WR and SC capacities decreased significantly with declining SPEI values, indicating that drought conditions impaired the ability of the forest to provide these services. The CS was less affected by drought, while the CS capacity of cropland responded relatively quickly to drought (Figure 3A). During the 2022 drought event, both cropland and forest experienced substantial decreases in CS, WR, and SC capacities, alongside a notable reduction in FS of cropland (Figure 3B). The analysis also revealed that FS maintained relatively high levels even during drought years. Comparative analysis showed that, except for CS, the WR, SC, and FS values during drought periods were considerably lower than those during non-drought periods, with WR and SC displaying the most substantial decreases (Figure 3C).

**Figure 4:**
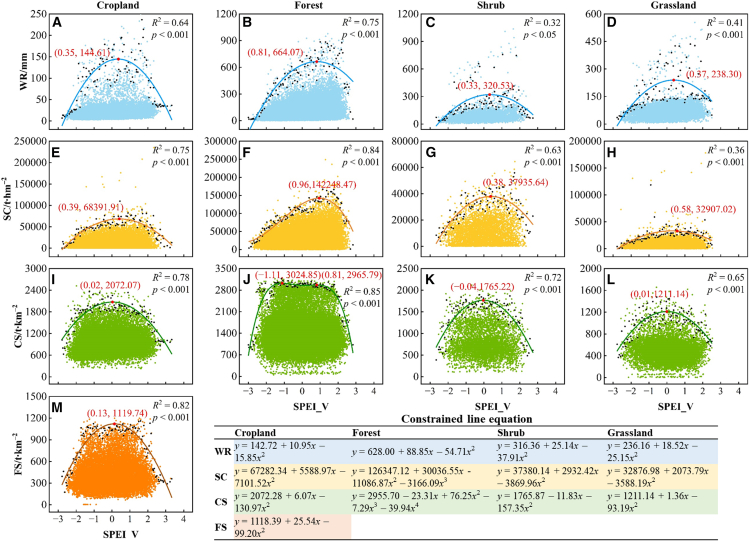
Constraint lines and equations between SPEI and ESs Panels (A–M) show the types of constraint lines and their threshold points between ESs and SPEI across different land use types.

Constraint lines between SPEI and ESs were established across distinct land use types, with corresponding thresholds identified (Figure 4). Excluding the convex-waved curve relationship between SPEI and CS in the forest ecosystem, SPEI and ESs exhibited hump-shaped curves in all other land-use types. This indicates a threshold effect of drought on ESs, with response patterns regulated by land use. Specifically, the drought response thresholds for WR and SC in forests reached 0.81 and 0.96, respectively, both higher than those under other land-use types. Furthermore, SPEI values at most threshold points exceeded −0.5, demonstrating that intensified drought generally diminishes ES supply capacity.

### Impacts of different levels of drought on ecosystem services

**Figure 5:**
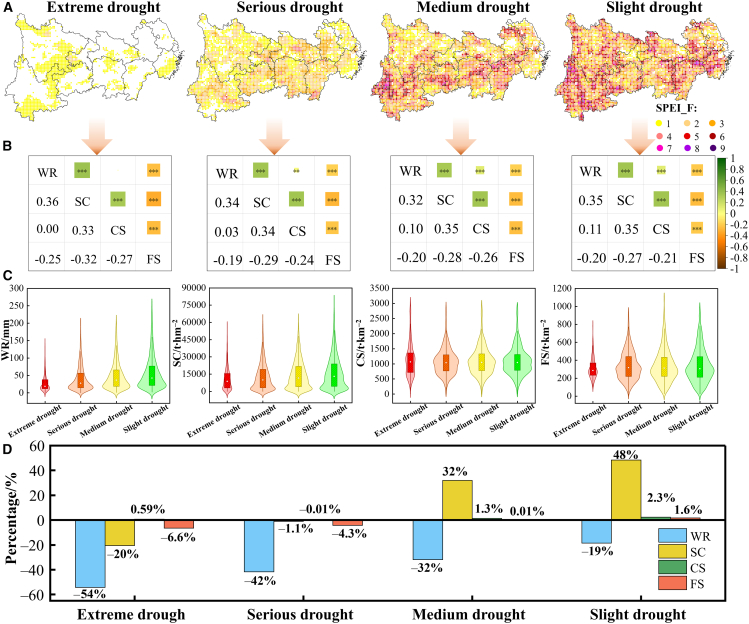
ESs and their relationships under different drought degrees (A) Frequency of extreme, serious, medium, and slight drought in the YREB from 2000 to 2022. (B) Trade-offs and synergies relationships among ESs under different drought degrees. (C) Statistical distributions of four ESs under different drought degrees. The violin plots depict the kernel density estimation of the data distribution, with overlaid boxplots showing the median (white dots), the 25%–75% interquartile range (IQR, box limits), and the 1.5 × IQR (whiskers). (D) Comparison of the changes in ESs in the event of different drought degrees. SPEI_F, drought frequency; YREB, Yangtze River Economic Belt. The same below.

Figure 5A presents the drought severity classification and frequency statistics from 2000 to 2022. Slight drought events occurred across almost the entire YREB, with Yunnan having the highest proportion of areas experiencing multiple slight droughts. Medium drought frequency was higher in southern YREB. Serious and extreme droughts were less frequent, with extreme drought events being virtually absent in Jiangsu and Anhui. ES trade-offs and synergies differed under varying drought severities (Figure 5B). Pearson correlation analysis revealed trade-offs and synergies among ESs, with synergistic relationships observed between WR and SC, WR and CS, and SC and CS, whereas FS showed trade-offs with the other three ESs (i.e., WR, SC, and CS). Notably, the strongest trade-offs emerged between WR and FS (*R* = −0.25, *p* < 0.001) and between SC and FS (*R* = −0.32, *p* < 0.001) in extreme drought zones. However, slight drought areas exhibited the strongest synergy between WR and CS (*R* = 0.11, *p* < 0.001). Overall, extreme drought areas had the lowest ES values, whereas CS and FS remained relatively stable across drought conditions (Figure 5C). Most ES losses intensified with drought severity (Figure 5D), whereas SC demonstrated enhanced capacity under slight and medium droughts.

**Figure 6:**
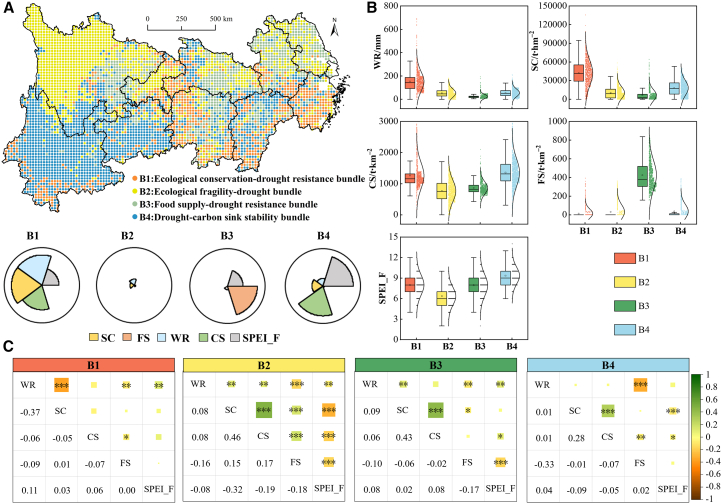
Composition of ES bundles and the trade-offs and synergies of ESs within bundles (A) Composition of ES bundles and their spatial distribution. (B) Distribution of ES values within different bundles. The boxplot represent the median (center line), the 25%–75% IQR (box limits), and the 1.5 × IQR (whiskers), with a square indicating the mean and an overlaid normal curve fitted to the data’s mean and SD. (C) Differences in trade-offs and synergies relationships among ESs under different bundles.

The YREB was partitioned into four ES bundles at the grid scale based on ESs and drought frequency (SPEI_F; Figure 6A): Ecological conservation-drought resistance bundle (B1), Ecological fragility-drought bundle (B2), Food supply-drought resistance bundle (B3), and Drought-carbon sink stability bundle (B4). B1 was predominantly distributed in the midstream and downstream areas of the YREB, while B2 primarily covered western Sichuan. B3 was widely distributed in major agricultural production zones such as the Sichuan Basin and Dongting Lake Plain, and B4 dominated most areas of Yunnan and Guizhou. Figure 6B illustrates the distribution of ESs and SPEI_F across bundles. B1 exhibited the strongest WR and SC capacities, with CS second only to B4, but its FS capacity was notably weak. B3 exhibited the highest FS capacity, yet demonstrated comparatively weaker WR and SC performance. SPEI_F analysis revealed that B4 experienced the highest drought occurrence, while B2 had the lowest. Analysis of trade-offs and synergies within each bundle showed that WR and SC exhibited trade-off relationship (*R* = −0.37, *p* < 0.001) in B1 but weak synergies in other bundles (Figure 6C). Notably, B2 displayed significant trade-offs between SPEI_F and all ESs (*p* < 0.01), while SC and CS showed the strongest synergy (*R* = 0.46, *p* < 0.001). Both B3 and B4 exhibited trade-off relationships between FS and the other ESs (i.e., WR, SC, and CS), with the strongest trade-off occurring between FS and WR.

### Analysis of the interactive effects of drought and other drivers on ecosystem services

**Figure 7:**
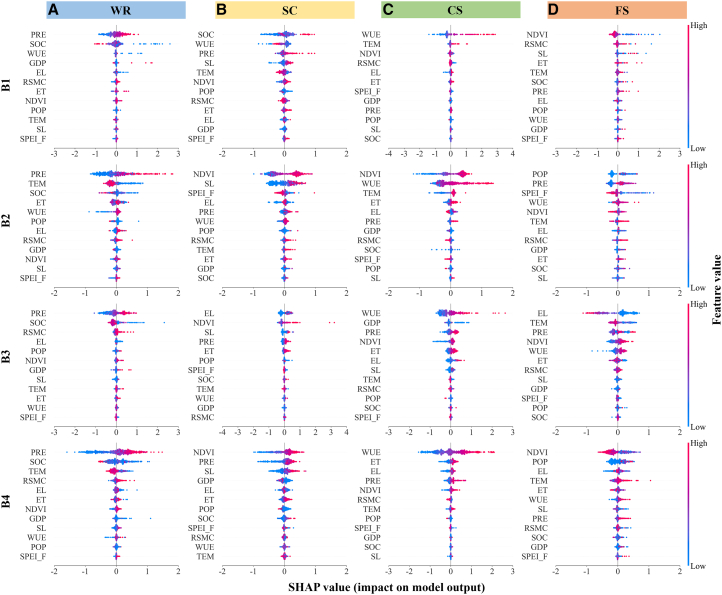
SHAP interpretation of ESs across distinct ES bundles (A), (B), (C), and (D) represent the SHAP values and feature importance of various driving factors for WR, SC, CS, and FS within bundles B1, B2, B3, and B4, respectively. EL, elevation; ET, actual evapotranspiration; GDP, gross domestic product; NDVI, normalized difference vegetation index; POP, population density; PRE, precipitation; RF, random forest; RSMC, relative soil moisture content; SHAP, Shapley additive explanations; SL, slope; SOC, soil organic carbon; TEM, temperature; WUE, water use efficiency. The same below.

Figures 7 and S2 present the analysis of Shapley additive explanations (SHAP) values within each ES bundle. Across all bundles, precipitation exerted positive effects on WR, with higher feature values corresponding to increased SHAP values. For SC, higher SHAP values occurred under high normalized difference vegetation index (NDVI) and soil organic carbon (SOC). Water use efficiency (WUE) and NDVI were critical drivers of CS, with elevated SHAP values observed at high WUE and NDVI. In B3, elevation was the most influential factor (|SHAP| value = 0.37) for FS, where lower elevation corresponded to higher SHAP values, indicating the negative impact of elevation on FS. SPEI_F generally showed negative impacts on ESs in most bundles, particularly on SC and FS in B2.

**Figure 8:**
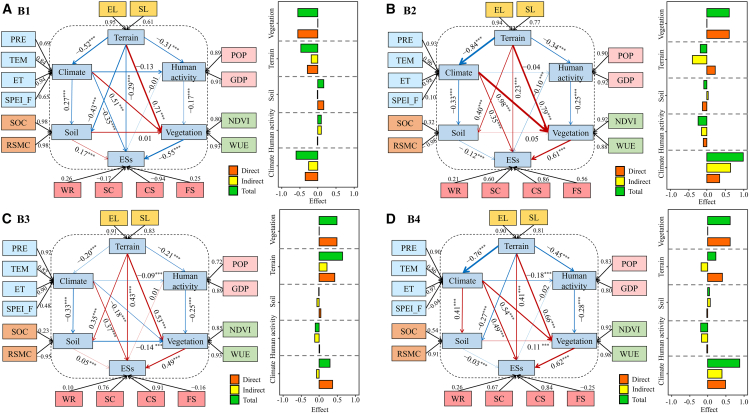
Partial least squares structural equation modeling of factors influencing ESs Red arrows indicate positive effects; blue arrows denote negative effects; line thickness corresponds to effect magnitude. Black arrows represent factor loadings of observed variables under each latent variable.

The partial least squares structural equation modeling (PLS-SEM) clarified the integrated impacts of climate, terrain, vegetation, soil, and human activity on ESs (Figure 8). In B1, terrain, climate, and vegetation exerted negative direct effects on ESs, contrasting with positive direct effects on B2, B3, and B4. Within B2, lower loading of relative soil moisture content (RSMC) intensified the soil’s negative impact on the ESs (path coefficient = −0.12, *p* < 0.001). Human activities exerted mostly negative effects on ESs across bundles. Among climatic indicators, SPEI_F demonstrated lower loading compared to that of other factors, except in B1, where its higher loading amplified the negative impacts of climate on ESs (path coefficient = −0.35, *p* < 0.001).

## Discussion

### Impact of drought on ecosystem services and their relationships

Under climate change, it is imperative to recognize that drought impacts ESs, as these directly affect societal sustainability. By altering ecosystem structures and processes, drought may degrade services such as water provisioning and crop yields.^33^ This study revealed that drought indeed weakened the supply of ESs in the study area, particularly reducing the WR and SC. However, the degradation of one ES often cascades to others, exacerbating trade-offs among ESs.^34^ For instance, our results showed that drought intensifies trade-offs between FS and three other services (i.e., WR, SC, and CS). Previous studies corroborate that drought intensifies the trade-off relationships between biodiversity preservation and forest ESs.^35^ Therefore, the consequences of drought on ESs demand urgent attention.

However, drought responses vary across ecosystems, likely due to differences in ES types and drought severity.^28^ Previous studies confirmed that drought imposes greater growth constraints on woody vegetation than on grasslands in water-limited regions,^36^ aligning with our findings. Extreme drought areas revealed the strongest synergy between SC and WR. Combined with the observed maximal reductions in these services during extreme droughts, this indicates their concurrent degradation. Such patterns likely stem from drought conditions limiting vegetation growth,^37^ subsequently impairing WR and SC capacities.

Moreover, this study found a relatively minor impact of drought on CS, which may be attributed to the regulatory effects of soil moisture and the adaptive capacity of ecosystems partially offsetting the influence of drought on CS.^38^ In fact, the impact of drought on carbon cycling depends on ecosystem status and prior climatic conditions. Water deficits during periods conducive to vegetation growth initially stimulate biomass accumulation, thereby further depleting soil moisture and increasing drought risks.^39^ Additionally, mixed-species forests have been found to maintain relatively high CS levels after experiencing extreme climatic events such as droughts.^40^ However, human interventions can exacerbate or mitigate drought impacts on ESs. This study observed that before 2004, droughts significantly affected FS, with marked reductions in grain yields during drought events. This is probably due to the adverse impacts of droughts on crop growth and development, thereby reducing photosynthesis and productivity.^41^ However, in recent years, FS has shown less susceptibility to drought, maintaining high FS capacity even under drought conditions. This improvement may be attributed to enhanced agricultural infrastructure, where irrigation practices alleviate meteorological drought in agroecosystems, ensuring stable food provision.^42^

### Coupling effects between drought and other drivers

Our integrated analysis of multiple ESs revealed that the key influencing factors were primarily precipitation, NDVI, WUE, and elevation, consistent with findings from Yang et al.^43^ and Fang et al.^44^ Under conditions of higher precipitation, NDVI, and WUE, the corresponding WR, SC, and CS capacities were significantly enhanced. However, FS capacity was weaker in high-elevation areas. Moreover, PLS-SEM demonstrated that under critically low RSMC conditions (B2), the soil exerted the most substantial negative impact on ESs. This occurs primarily because critically low RSMC restricts root water uptake by limiting direct water supply to the vegetation, thereby exacerbating ecosystem vulnerability.^45^ Meanwhile, human activity predominantly exerts negative effects on ESs, with excessive anthropogenic disturbance leading to marked declines in ESs.^46^ Although SPEI_F exerted specific impacts on ES supply, random forest (RF) analysis of driving factors ranked its relative importance lower. This phenomenon likely originates from ecosystem resilience, defined as the capacity to recover from disturbances such as drought, where systems exhibit negligible or minimal adverse impacts when disturbed, thus maintaining ecological stability.^47^

Variations in ESs are influenced by multiple factors, and interactions among these driving factors can indirectly affect ES supply.^48^ For instance, PLS-SEM revealed that in B2, B3, and B4, terrain exerted direct positive effects on overall ESs while generating indirect negative impacts through climatic influences. This aligns with findings from Zhang et al.^49^ and Li et al.,^50^ demonstrating that naturally dominated ESs are mainly influenced by terrain and climate. Among climatic observational variables, analysis revealed that B1 showed higher SPEI_F loadings, under which climatic impacts demonstrated the strongest negative effects on ESs. Furthermore, vegetation displayed greater path coefficients than other latent variables, potentially because of the role of vegetation as the primary producer in ecosystems, where variations in species composition and growth states directly drive spatial heterogeneity in ES supply, thereby exerting a critical regulatory effect.^51^ Vegetation growth dynamics are closely linked to climatic conditions. Drought-induced water deficits, for instance, impair vegetation development and alter phenological phases, with severe cases leading to mortality.^52^

The influence intensity of driving factors on ESs also relates to time scales. Studies have demonstrated that drivers of ES bundles vary across time scales, indicating that factors affecting ES supply and interactions depend on specific environmental contexts.^53^ In this study, RF and PLS-SEM were employed to investigate the long-term impacts and relative importance of driving factors. Thus, although drought ranked lower in relative importance among drivers for certain ESs, it still poses a significant barrier to the sustainable provision of ESs. The synergistic effects between drought and other drivers may amplify impacts on ESs.^54^ The thresholds of the constraint lines further demonstrated that the impact of drought on ESs was related to site conditions and varied across land-use types. These divergences primarily stem from differences in WUE and ecosystem resilience among forests, shrubs, grasslands, and croplands.^55^ Consequently, governmental authorities could establish early-warning systems based on these thresholds, proving crucial for predicting and mitigating climate change impacts on ecosystems.

These findings deepen our understanding of the complex response mechanisms of ESs in the YREB under climate change and also provide a scientific basis for ecosystem sustainability planning and the rational utilization of land resources at both the regional level and globally.

### Zoning management strategies for ecosystem service bundles

Based on the response characteristics of ES bundles to drought and interactions between driving factors, we proposed targeted recommendations for spatial planning and management.

(1) Ecological conservation-drought resistance bundle (B1): strengthen the protection of vegetation in mountainous and hilly areas, and promptly conduct the ecological restoration of vegetation damaged by drought; establish a monitoring network integrating remote sensing and ground observations to enhance the early warning and dynamic tracking of extreme weather such as drought.
(2) Ecological fragility-drought bundle (B2): strengthen water resource protection and enhance natural water storage and conservation capabilities; demarcate control red lines for ecologically fragile areas and restrict unreasonable human activities.
(3) Food supply-drought resistance bundle (B3): promote the construction of high-standard farmland equipped with precision irrigation systems to improve water use efficiency; implement straw return-to-field, organic fertilization, and other cultivation management measures to improve the water retention and fertilizer retention capacity of the soil.
(4) Drought-carbon sink stability bundle (B4): optimize water resource allocation by improving water storage facilities and promoting water saving measures; enhance the stability of forest carbon sinks through initiatives including the Grain-for-Green Program and natural forest conservation projects.

### Limitations of the study

This study has several limitations. First, considering that the YREB experienced rapid economic development around 2000, with ESs exhibiting distinct variation characteristics.^56^ Before 2000, high-resolution ecological remote sensing data were relatively difficult to obtain; therefore, we selected the period 2000–2022 for this study. Previous studies have shown that this period covers multiple typical drought events in the study area.^57^ However, the 2000–2022 analysis period falls short of the 30-year climatological standard for meteorological baselines. This temporal constraint may restrict the comprehensive assessment of long-term ES trajectories and drought cumulative effects. Future studies incorporate extended time series to better evaluate the drought variability characteristics and ES impact mechanisms.^58^ Additionally, the integration of CMIP6 climate model projection data helped simulate drought intensity and frequency trends under multiple scenarios, improving predictions of drought impacts on ES supply capacity and bundle relationships under climate change.

Second, due to the spatiotemporal resolution limitations of input data and process simplifications in the ES assessment model, this study cannot reliably generate monthly scale spatial distribution data of ESs. In particular, it is hard to characterize the differences in drought responses of FS during critical crop growth stages, resulting in insufficient analysis of the differential impact mechanisms of seasonal drought on key ESs.^59^ In addition, we could not fully capture potential ecological mechanisms such as vegetation stomatal regulation and deep root adaptation. Future research can improve the accuracy of the study by coupling physical process models with deep learning algorithms as well as combining remote sensing and ground observation data with higher spatiotemporal resolution.

Finally, the YREB has a subtropical monsoon climate, where the combination of abnormal precipitation and high-temperature events can easily induce meteorological drought.^60^ Therefore, meteorological drought is a key drought type affecting regional ESs. Considering the direct impact of agricultural drought on ecosystems, we used the RSMC indicator in the driving factors to preliminarily reveal the impact of agricultural drought on ESs. In addition, due to the dense river networks and strong reservoir regulation capacity, this region has abundant surface water resources, which can mitigate runoff deficits to a certain extent. However, the effect of hydrological drought cannot be entirely neglected. Future research should integrate multiple types of drought indicators for comprehensive analysis,^61^ and explore the changes in trade-offs and synergistic relationships among ESs under compound drought scenarios.

## Resource availability

### Lead contact

Further information and requests for resources should be directed to and will be fulfilled by the lead contact, Wei Wan (wanwei@ncu.edu.cn).

### Materials availability

This study did not generate new unique materials.

### Data and code availability

• This article analyses existing publicly available data. The data sources are listed in the key resources table.
• All original code can be available on request from the lead contact.
• Any additional information required to reanalyze the data reported in this article is available from the lead contact upon request.

## Acknowledgments

This work was supported by the 10.13039/501100004479Natural Science Foundation of Jiangxi Province (Grant No. 20252BAC200623), the 10.13039/501100001809National Natural Science Foundation of China (Grant No. 42301091), the 10.13039/501100013064Key Research and Development Program of Jiangxi Province (Grant No. 20223BBG74S01; No. 20223BBG71013), and the Innovation Fund Designated for Graduate Students of Jiangxi Province (Grant No. YC2025-B047).

## Author contributions

Liujie He: writing – original draft, conceptualization, methodology, visualization, investigation, formal analysis, and data curation. Shuyang Wu: data curation, methodology, and validation. Zeyang Xie: formal analysis and data curation. Han Liang: formal analysis and investigation. Zhijian Wu: methodology an investigation. Deli Xiao: methodology and data curation. Jinqi Zhu: resources and software. Bofu Zheng: validation, writing – review and editing, methodology, and project administration. Wei Wan: validation, methodology, project administration, and writing – review and editing. All authors reviewed the article.

## Declaration of interests

The authors declare no competing interests.

## STAR★Methods

### Key resources table

**Table:** 

REAGENT or RESOURCE RESOURCE	SOURCE	IDENTIFIER
**Deposited data**		

Land use (30 m; 2000—2022)	Earth System Science Data	https://essd.copernicus.org/
Topographic data (30 m; /)	Geospatial Data Cloud	http://www.gscloud.cn/
Soil texture and soil organic carbon (1 km; /)	National Tibetan Plateau Data Center	https://data.tpdc.ac.cn/
Soil moisture (1 km; 2000—2022)	National Tibetan Plateau Data Center	https://data.tpdc.ac.cn/
Standardized precipitation evaporation index (5 km; 2000—2022)	Earth System Science Data	https://essd.copernicus.org/
Potential evapotranspiration (1 km; 2000—2022)	National Tibetan Plateau Data Center	https://data.tpdc.ac.cn/
Actual evapotranspiration (5 km; 2000—2022)	Climatology Lab	https://www.climatologylab.org/
Precipitation (1 km; 2000—2022)	National Tibetan Plateau Data Center	https://data.tpdc.ac.cn/
Temperature (1 km; 2000—2022)	National Tibetan Plateau Data Center	https://data.tpdc.ac.cn/
Normalized difference vegetation index (1 km; 2000—2022)	Resources and Environmental Sciences Data Platform	https://www.resdc.cn/
Net primary production (1 km; 2000—2022)	National Aeronautics and Space Administration	https://www.nasa.gov/
Gross primary productivity (5 km; 2000—2022)	National Aeronautics and Space Administration	https://www.nasa.gov/
Grain yield (/; 2000—2022)	Statistical Yearbook	https://navi.cnki.net/knavi/
Population density (1 km; 2000—2022)	Oak Ridge National Laboratory	https://landscan.ornl.gov/
Gross domestic product (1 km; 2000—2022)	Scientific Data	https://www.nature.com/

**Software and algorithms**		

MATLABR2024a	MathWorks	https://www.mathworks.com/
Origin 2021	OriginLab	https://www.originlab.com/
ArcGIS	Environmental Systems Research Institute	https://www.arcgis.com/
R Project 4.4.1	R Foundation	https://www.r-project.org/
Python 3.7	Python Software Foundation	https://www.python.org/

The content in parentheses in the first column of the table indicates the spatial resolution (first value) and time span (second value) of the data, “/” indicates that there is no spatial resolution or time span.

### Method details

#### Study region

**Figure 9:**
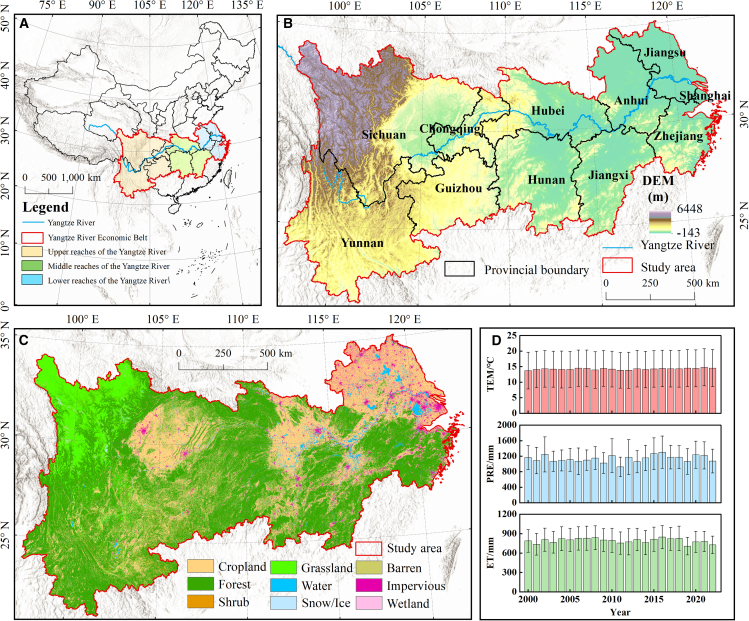
Profile of the study region (A) Geographical position and watershed delineation (upper, middle, lower reaches) of the YREB. (B) Digital elevation model (DEM) and provincial administrative boundaries. (C) Spatial distribution of land use types. (D) Interannual variations of mean annual TEM, PRE, and ET during 2000–2022. Data are represented as mean ± SD.

The YREB spans eastern, central, and western China, covering 11 provinces and municipalities with a total area of approximately 2.05 million km^2^ (Figure 9). As the most dynamic region for China’s socio-economic development, the YREB generated 46.7% of gross domestic product (GDP) in 2023. Characterized by dense river networks and abundant water resources, the region also faces soil erosion risks due to its steep terrain. With high vegetation coverage, its forest ecosystems exhibit significant carbon sequestration advantages. Simultaneously, as China’s critical food production base, the YREB contains about one-quarter of the country’s cultivated land and accounts for 40% of national agricultural output. Consequently, WR, SC, CS, and FS are the YREB’s typical ESs. Most areas in the YREB experience a subtropical monsoon climate, with mean annual temperature ranging from 14 to 16°C and yearly precipitation between 800 and 1400 mm. This region exhibits characteristic concurrent precipitation and thermal regimes, where abnormally reduced rainfall or elevated temperature significantly predisposes the area to meteorological drought occurrences.^62^ Therefore, it is representative and typical to explore the impacts and mechanisms of meteorological drought on ESs in the YREB.

#### Data sources and pre-processing

The data sources used in this study are presented in key resources table, including land use, meteorological, soil, vegetation, topographic and socioeconomic data. WUE was calculated as the ratio of gross primary productivity to actual evapotranspiration (Figure S3).^53^ The drought level classification of the SPEI was presented in Table S1.^63^ All raster data were resampled to a spatial resolution of 1 × 1 km using the bilinear interpolation method.

#### Research framework

**Figure 10:**
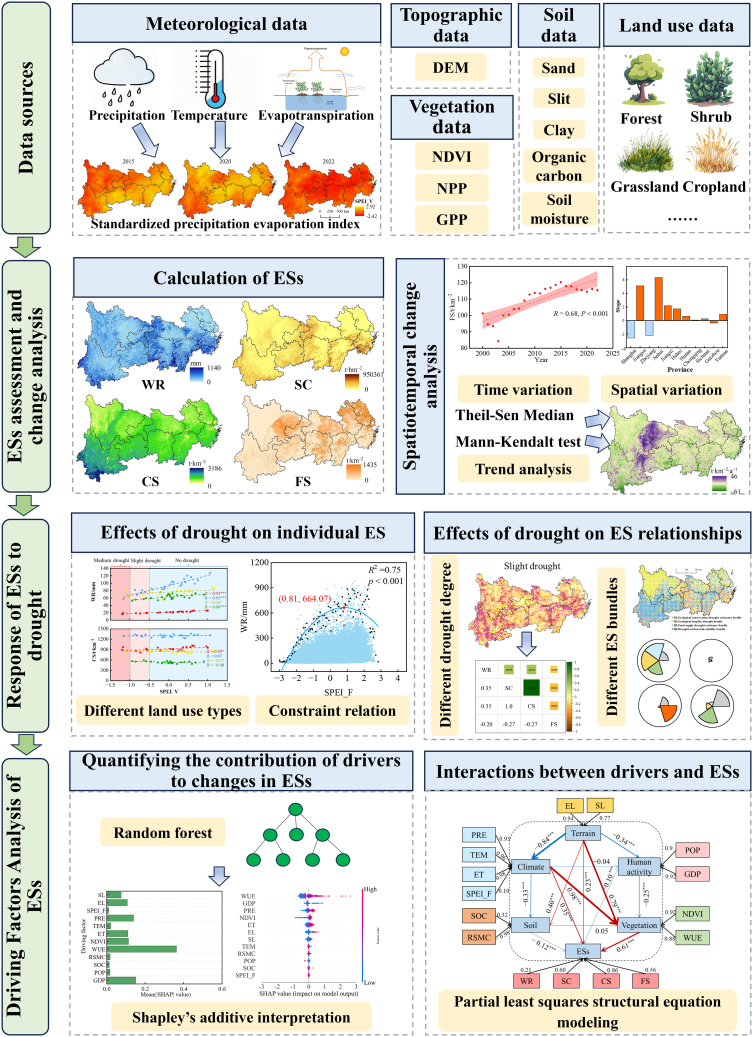
Conceptual framework of the study

The research framework of this study (Figure 10), includes three parts: the assessment and changes of ESs; response of ESs to drought; and analysis of driving factors influencing ESs.

#### Evaluation of ecosystem services

Synthesizing existing research and the YREB’s distinct geographic and socioeconomic characteristics,^64^^,^^65^ this study focuses on four key ESs for assessment: WR, SC, CS, and FS, with specific evaluation methods for each indicator detailed in Table 1.

**Table 1:** Calculation method for ecosystem services (ESs)

ESs	Principles	Mathematical expression
Water retention (WR)	Guided by the water balance principle, this study employed the InVEST model to compute water yield, integrating runoff, topography, saturated hydraulic conductivity, and water yield in the analytical framework.^66^	$Retention=\min\left(1,\frac{249}{Velovity}\right)\times \min\left(1,\frac{0.9\times TI}{3}\right)\times \min\left(1,\frac{K_{s}}{300}\right)\times Yield$ Where *Retention* represents WR, mm; *TI* denotes the topographic index, dimensionless; *K*_s_ is saturated hydraulic conductivity, cm/d; *Velocity* indicates the flow velocity coefficient, dimensionless; *Yield* corresponds to calculated water yield, mm.
Soil conservation (SC)	SC was quantified using the Revised Universal Soil Loss Equation.^67^	*Q*_sc_ = *R*×*K*×*L*×*S*×(1-*P*×*C*) Where *Q*_sc_ represents SC capacity t·(hm^2^·a)^−1^; *R* denotes the precipitation erosion factor MJ·mm·(hm^2^·h·a)^−1^; *C* is the vegetation cover factor, dimensionless; *L* indicates the slope length factor, dimensionless; *S* corresponds to the slope factor, dimensionless; *K* represents the soil erodibility factor, t·h·(MJ·mm)^−1^; *P* signifies the soil and water conservation measure factor, dimensionless.
Carbon sequestration (CS)	CS was quantified using CO_2_ uptake from vegetation photosynthesis, estimated via net primary productivity (NPP).^68^	*G*_*V*_ = 1.63×*A*×*NPP* Where *G*_*v*_ represents vegetation CS, g·a^−1^; *A* denotes ecosystem area, km^2^.
Food supply (FS)	Grain yield data at the municipal administrative level were spatially allocated to cropland grid cells based on the proportional distribution of normalized difference vegetation index (NDVI) values.^69^	*P*_*i*_ = NDVI_*i*_/NDVI_sum_×*P*_sum_ Where *P*_*i*_ represents grain yield of the *i*-th pixel, t/pixel; *P*_sum_ denotes total grain yield at the municipal level, t; *NDVI*_*i*_ is the NDVI value of the *i*-th cropland pixel, dimensionless; *NDVI*_sum_ corresponds to the sum of NDVI values across municipal croplands, dimensionless.

#### Identification of ecosystem service bundles

The self-organizing map (SOM), an unsupervised neural network algorithm, combines dimensionality reduction and clustering capabilities to spatially classify grid cells into ES bundles based on spatial similarity. This method is applicable to different spatial scales.^70^ This study implemented SOM using the “kohonen” package in R 4.4.1.^53^ To address unit heterogeneity among the four ES indicators, z-score standardization was applied to normalize all variables prior to analytical processing.

#### Construction of constraint line

This study employed the quantile segmentation method to extract constraint lines, characterizing the distribution boundaries or potential maxima of response variables under constraining factors, effectively delineating the impact processes of dominant driving factors on these variables.^8^ The implementation procedure consisted of: (1) dividing constraining factors into 100 equal intervals based on their numerical values; (2) extracting the 99.9^th^ percentile within each interval as boundary points; (3) fitting these points through regression, with constraint lines determined by scatterplot morphology and maximum goodness of fit (*R*^2^; Figure S4).^71^ Thresholds were determined through derivative equation analysis of constraint lines at inflection points in constraint-effect relationships between variables.^64^

#### Random forest modeling and shapley additive explanations

This study employed RF and SHAP models to analyze driving factors of ESs. As a non-parametric ensemble learning method, RF quantifies the contribution of individual factors to ES variations and demonstrates superior predictive accuracy among machine learning algorithms.^72^ The model effectively captures nonlinear relationships and complex interaction effects in data, with enhanced resistance to overfitting and robust performance.^73^ SHAP analysis complements RF’s limitations in feature interpretability by assessing positive/negative impacts of independent variables on responses, thereby elucidating interaction mechanisms between ESs and their driving factors. SHAP results were generated using the “shap” package in Python 3.7.^74^ The Shapley value calculation for each feature follows.

(Equation 1) $$\phi_{i}=\sum_{S\subseteq U\setminus \left\{i\right\}}\frac{\left|S\right|!\left(\left|N\right|-\left|S\right|-1\right)!}{\left|N\right|!}\left[f_{x}\left(S\cup \left\{i\right\}\right)-f_{x}\left(S\right)\right]$$

where *ϕ*_*i*_ represents the Shapley value of the *i*-th feature; *N* denotes the set of all features; *S* is the subset *N* excluding the *i*-th feature; *f*_*x*_(*S*∪{*i*}) indicates the model output including the *i*-th feature; and *f*_*x*_(*S*) corresponds to the model output excluding the *i*-th feature.

#### Structural equation model

This study utilized PLS-SEM to assess complex relationships between driving factors and ESs. The model is less susceptible to multicollinearity and missing data issues,^48^ comprising two components: an inner model (defining relationships among latent variables) and an outer model (linking latent variables to observed indicators).^75^ Based on prior research,^22^^,^^70^ we selected six latent variables: terrain, climate, vegetation, soil, human activity, and ESs. For the observation variables, we chose four ESs (i.e., WR, SC, CS, and FS), along with twelve driving factor indicators: temperature, precipitation, actual evapotranspiration, SPEI_F, elevation, slope, NDVI, WUE, SOC, RSMC, GDP, and population density. To address unit heterogeneity, all variables were standardized using the Z-score method prior to model construction. This study constructed the PLS-SEM based on a comprehensive conceptual framework and all models exhibited Goodness-of-Fit indices exceeding 0.6, indicating reliable models and valid results.^76^ All analyses were conducted using R 4.4.1,^77^ and the PLS-SEM was implemented with the “plspm” package.

### Quantification and statistical analysis

Quantification and statistical analysis are mentioned in the method details section.
