# Supplementary material for: Drought diminishes ecosystem service supply and exacerbates trade-offs in the Yangtze River Economic Belt
Source: iScience. 2025 Sep 19;28(10):113604. doi: 10.1016/j.isci.2025.113604 (PMC12539323; doi:10.1016/j.isci.2025.113604)
Supplement: Document S1. Figures S1–S4 and Table S1 [file mmc1.pdf]

iScience, Volume *28*

Supplemental information

Drought diminishes ecosystem service supply and exacerbates trade-offs in the Yangtze River Economic Belt

Liujie He, Shuyang Wu, Zeyang Xie, Han Liang, Zhijian Wu, Deli Xiao, Jinqi Zhu, Bofu Zheng, and Wei Wan

Supplemental information

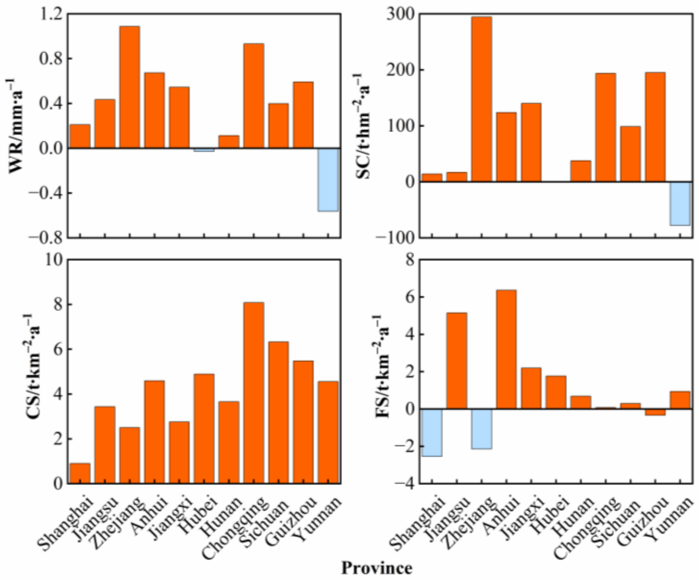

Figure S1 Sen values of ecosystem services in different provinces

Notes: WR, water retention; SC, soil conservation; CS, carbon sequestration; FS, food supply. The same below.

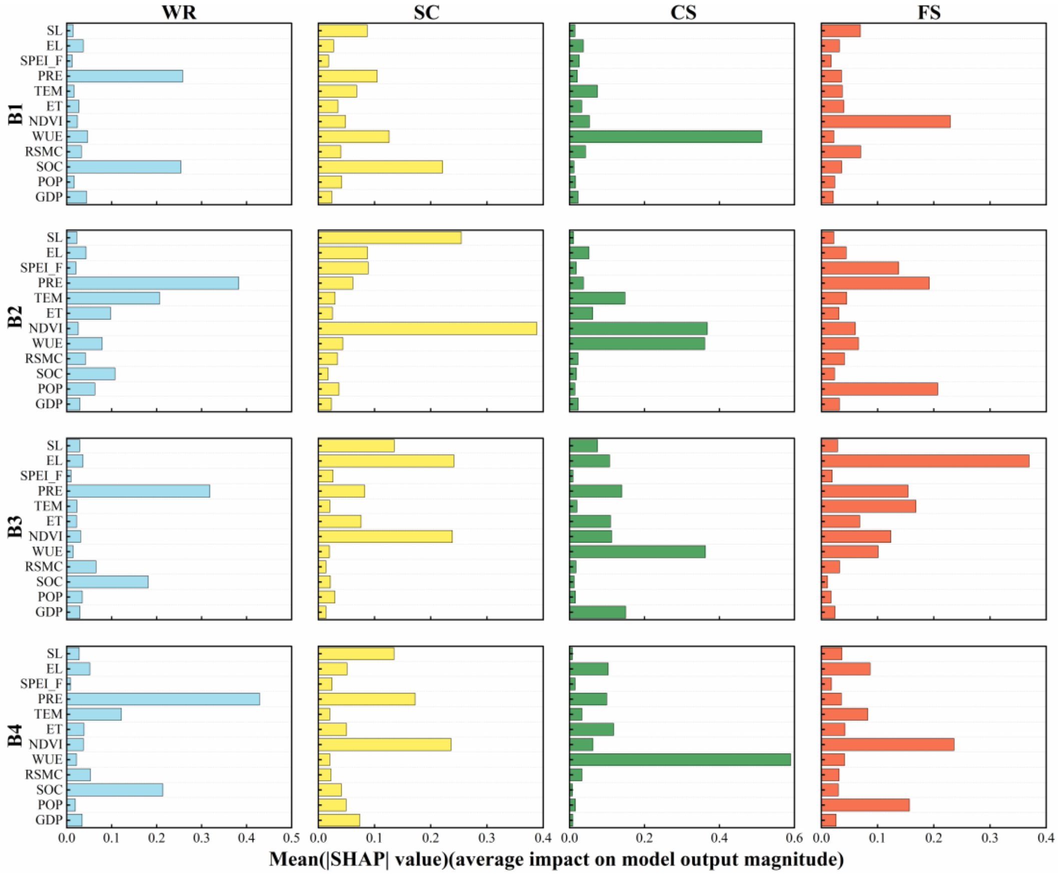

Figure S2 Mean SHAP values for each ecosystem service driver

Notes: SHAP, Shapley additive explanations.

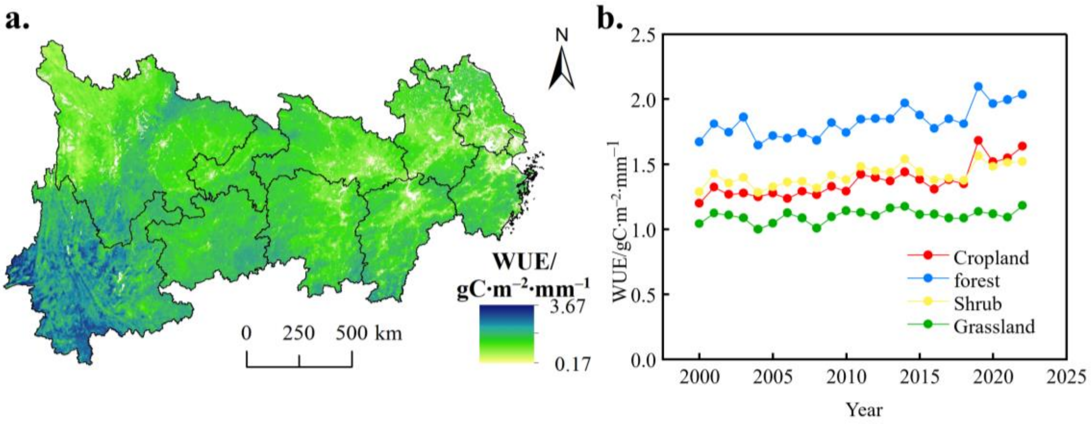

Figure S3 Spatiotemporal distribution characteristics of water use efficiency and its variations across land use types.

Notes: WUE, water use efficiency.

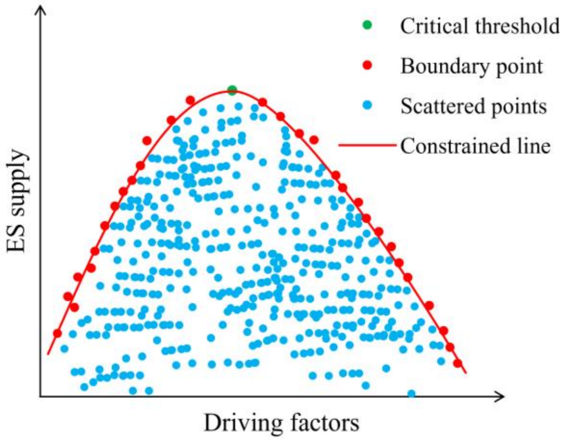

Figure S4 Schematic diagram of the constraint line

Table S1 Classification scales of drought indices

Drought severity	Meteorological drought
No drought	SPEI > −0.5
Slight drought	−1.0 < SPEI ≤ −0.5
Medium drought	−1.5 < SPEI ≤ −1.0
Serious drought	−2.0 < SPEI ≤ −1.5
Extreme drought	SPEI ≤ −2.0
